# Supplementary material for: Clinical Importance of Regimens in Hepatic Arterial Infusion Chemotherapy for Advanced Hepatocellular Carcinoma with Macrovascular Invasion
Source: Cancers (Basel). 2021 Sep 3;13(17):4450. doi: 10.3390/cancers13174450 (PMC8431395; doi:10.3390/cancers13174450)
Supplement: Supplementary file 1 [file cancers-13-04450-s001.zip › cancers-1320856-supplementary.pdf]

cancers MDPI

Supplementary Materials: Clinical Importance of Regimens in Hepatic Arterial Infusion Chemotherapy for Advanced Hepatocellular Carcinoma with Macrovascular Invasion

Takashi Niizeki [1], Hideki Iwamoto [1,2,*], Tomotake Shirono [1], Shigeo Shimose [1], Masahito Nakano [1], Shusuke Okamura [1], Yu Noda [1], Naoki Kamachi [1], Hiroyuki Suzuki [1], Miwa Sakai [1], Ryoko Kuromatsu [1], Hironori Koga[1] and Takuji Torimura [1]

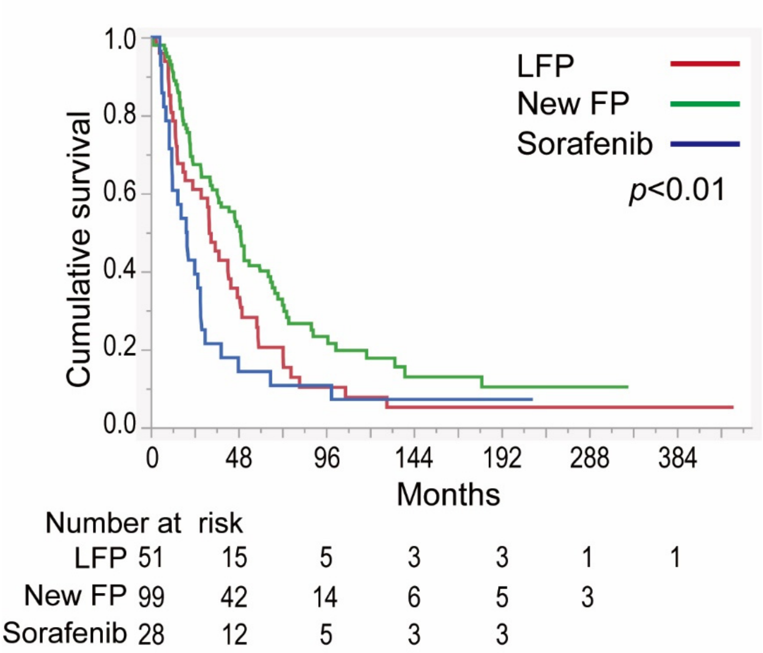

Figure S1. Comparison of the survival curve sorafenib, LFP and New FP groups. The MST of sorafenib was 9.7 months. The LFP group was 16.1 months. The MST of New FP was 24.7 months. There were significant differences between two groups ($p < 0.01$).

Table S1. Patient and tumor characteristics of sorafenib group.

Items	Total (28)
Age	70.8 ± 9.9
Sex (male/female)	22/6
Etiology (HBV */HCV **/NBNC ***/ HBV+HCV)	6/20/2/0
Child-Pugh score (5/6)	15/13
WBC † (/μL)	5294 ± 1652
Plt †† (×10⁴/μL)	14.9 ± 6.8
AFP ††† (median) (ng/ml)	135.6 (1.9–720,500)
DCP ‡ (median) (mAU/ml)	2,397(14–75,000)
Maximum tumor diameter (mm)	63.9 ± 50.5
Tumor number single / multiple	5/23
Tumor occupancy (<3/≥3)	71/79
PVTT ‡‡ (trunk/ 1st or 2nd branch)	7/18
HVTT ‡‡‡ (present/ absent)	3/26
BDTT § (present/ absent)	1/27

* Hepatitis B virus, ** Hepatitis C virus, *** Alpha-phetoprotein, † Des-gamma-Carboxy Prothrombin, †† Portal vein tumor thrombus, ††† Alphaphetoprotein, ‡ Des-gamma-Carboxy Prothrombin, ‡‡ Portal vein tumor thrombosis, ‡‡‡ Hepatic vein tumor thrombosis, § Bile duct tumor thrombosis.

Table S2. Univariate and multivariate analysis of factors associate with poor survival in LFP.

Items	Uni Variate Analysis *p*-Value	Multivariate Analysis *p*-Value 95% CI
Age (≧ 70 years)	0.525	-
Gender (Male)	0.021	0.470
HBs * Antigen (+)	0.002	0.603
HCV ** Ab (−)	0.001	0.057
Child- Pugh score 6	0.213	-
Platelet count (≥11 × 10^4/μL)	0.004	0.194
AFP *** (≥400 ng/mL)	0.099	0.855
DCP † (≥1600 mAU/mL)	0.011	0.164
Maximum tumor diameter (≥70 mm)	0.003	0.521
Tumor located segments (≥3)	0.355	-
Grade of PVTT †† (trunk)	<0.001	<0.001 (0.217–1.138)
Hepatic vein tumor thrombus (+)	0.549	-

* Hepatitis B virus, ** Hepatitis C virus, *** Alpha-phetoprotein, † Des-gamma-Carboxy Prothrombin, †† Portal vein tumor thrombus.

Table S3. Univariate and multivariate analysis of factors associate with survival in New FP.

Items	Uni Variate Analysis P-Value	Multivariate Analysis *p*-Value 95% CI
Age (≥70 years)	0.101	0.667
Gender (Male)	0.043	0.470
HBs * Antigen (+)	0.624	-
HCV ** Ab (−)	0.969	-
Child-Pugh score 6	0.075	0.050
Platelet count (≥11 × 10^4 /μL)	0.235	-
AFP (≥400 ng/mL)	0.488	-
DCP (≥1600 mAU/mL)	0.049	0.121
Maximum tumor diameter (70mm)	0.724	0.483
Tumor located segments (≥3)	0.004	0.031 (0.538–0.26)
Grade of PVTT (trunk)	0.171	-
Hepatic vein tumor thrombus (+)	0.011	0.064

* Hepatitis B virus, ** Hepatitis C virus.
